# The benefits of adipocyte metabolism in bone health and regeneration

**DOI:** 10.3389/fcell.2023.1104709

**Published:** 2023-02-21

**Authors:** Lisa-Marie Burkhardt, Christian H. Bucher, Julia Löffler, Charlotte Rinne, Georg N. Duda, Sven Geissler, Tim J. Schulz, Katharina Schmidt-Bleek

**Affiliations:** ^1^ Julius Wolff Institute, Berlin Institute of Health (BIH) Charité, Berlin, Germany; ^2^ BIH Center for Regenerative Therapies (BCRT), Charité—Universitätsmedizin, Berlin, Germany; ^3^ Department of Adipocyte Development and Nutrition, German Institute of Human Nutrition, Potsdam-Rehbrücke, Nuthetal, Germany; ^4^ German Center for Diabetes Research (DZD), München-Neuherberg, Germany; ^5^ University of Potsdam, Institute of Nutritional Science, Nuthetal, Germany

**Keywords:** bone regeneration, immune cells, inflammation, adipocytes, bone marrow adipose tissue (BMAT), peroxisome proliferator-activated receptor γ (PPARG) agonists, mesenchymal stromal cells (MSCs), thiazolidinediones

## Abstract

Patients suffering from musculoskeletal diseases must cope with a diminished quality of life and an increased burden on medical expenses. The interaction of immune cells and mesenchymal stromal cells during bone regeneration is one of the key requirements for the restoration of skeletal integrity. While stromal cells of the osteo-chondral lineage support bone regeneration, an excessive accumulation of cells of the adipogenic lineage is thought to promote low-grade inflammation and impair bone regeneration. Increasing evidence indicates that pro-inflammatory signaling from adipocytes is responsible for various chronic musculoskeletal diseases. This review aims to summarize the features of bone marrow adipocytes by phenotype, function, secretory features, metabolic properties and their impact on bone formation. In detail, the master regulator of adipogenesis and prominent diabetes drug target, peroxisome proliferator-activated receptor γ (PPARG), will be debated as a potential therapeutic approach to enhance bone regeneration. We will explore the possibilities of using clinically established PPARG agonists, the thiazolidinediones (TZDs), as a treatment strategy to guide the induction of a pro-regenerative, metabolically active bone marrow adipose tissue. The impact of this PPARG induced bone marrow adipose tissue type on providing the necessary metabolites to sustain osteogenic-as well as beneficial immune cells during bone fracture healing will be highlighted.

## 1 Introduction: Bone-resident mesenchymal stromal cells and adipocytes

The osteogenic differentiation trajectory of mesenchymal stromal cells (MSCs) is crucial for bone tissue function, as they likely contribute to the formation of adult skeletal stem cells. For example, there are bone-resident populations of cells specialized to undergo the typical osteogenic maturation stages, including osteoblasts and osteocytes. Moreover, MSCs probably contribute significantly to chondrogenic lineages as a key component of endochondral ossification (Zhou et al., 2014; Bianco and Robey, 2015; Jones et al., 2016). The concerted actions of the MSC-derived progeny cells are key elements of embryonic bone formation, maintenance of adult bone homeostasis and are required for bone regeneration. Multipotent MSCs can undergo multi-lineage differentiation, pursuing pro-regenerative fates, such as osteogenic or chondrogenic differentiation, or adipogenic lineage specialization within the bone marrow compartment. The corresponding lineage commitment decision towards an adipocyte-fate depends on a multitude of factors making up the microenvironment in which the cell resides. This milieu includes distinct cytokines, growth factors, chemokines, neighboring cells, the underlying mechanical properties of the cell, oxygen tension and local metabolite concentrations. The specialization of MSCs towards an osteogenic fate is known to be essential for mineralized bone tissue formation, whereas adipogenic lineage commitment frequently occurs as a function of pathological processes, including aging and metabolic diseases (Pierce et al., 2019). Thus far, the literature consensus regarding the impact of bone marrow adipose tissue (BMAT) accumulation is that it exerts detrimental effects on bone health and regeneration. However, substantial evidence exists for an early onset of bone marrow adipogenesis in skeletal development, clearly suggesting that some quality of marrow adipogenesis may indeed be a normal physiological process or even a beneficial aspect of bone homeostasis. Accordingly, a number of studies have described contradictory observations regarding BMAT, but have also suggested that its role should be re-evaluated carefully with a more nuanced perspective. In this overview article, we will summarize the existing literature regarding potentially beneficial physiological roles of bone-resident adipocytes in bone homeostasis. We will also outline and discuss the know stimuli that promote marrow adipocyte formation and maintenance such that they may be employed to help sustain bone function and contribute to a rather pro-osteogenic microenvironment, i.e., we will evaluate the possibility whether different lineages of detrimental adipocytes may exist alongside regeneration-supportive subsets of adipocytic lineages and mature adipocytes.

However, due to the large heterogeneity of MSC populations, both in *in vitro* and *in vivo* experiments, there is no clear definition of MSCs in bone marrow (Brachtl et al., 2022). The MSC populations are very heterogeneous due to the tissues and niches they stem from, the purification strategy that has been applied and the number of passages they underwent during culturing. Their clonogenic potential is one of the hallmarks for their identification. Human bone marrow MSCs (hBM MSC) have been defined by various markers, and CD10, CD13, CD73, and CD105 are generally considered established markers (B̈uhring et al., 2007). However there is no finally defined marker set for their identification (Kouroupis et al., 2019). hBM MSCs are positive for CD271, a marker that is lost during culture expansion. CD146 marks hBM MSCs from the perivascular niche while CD 56 marks those that interact with osteoblastic cells. Nestin is another marker that is associated with perivascular hBM MSCs while CD56 is allocated to bone-lining cells (Kouroupis et al., 2019). More recently defined marker for hBM MSC are CD140b, MSCA-1, CD90, CD106, CD140a, and SUSD2 (B̈uhring et al., 2007; Busser et al., 2015; Mabuchi et al., 2013; Li et al., 2014).

Mouse models are relevant for pre-clinical studies of bone regeneration to characterize osteogenic and adipogenic MSC lineages and their function. Traditionally, the distinction of murine bone marrow MSCs from resident hematopoietic and endothelial cells includes the separation by expression of cell surface antigens such as CD29, CD90, CD44, CD81, CD106, CD24, Sca-1, and nucleostemin (Pittenger et al., 1999; Dominici et al., 2006). Further, negative selection of the following antigens: CD11b/integrin alpha M (exclusion of macrophages/monocytes), CD31/PECAM-1 (exclusion of endothelial cells), CD45 (exclusion of leukocytes), and CD117 and CD135 (exclusion of other hematopoietic/blood cells) is required (Pittenger et al., 1999; Dominici et al., 2006). Another frequently used marker is Sca-1, however it is only expressed in rodents but not humans (Peister et al., 2004; Houlihan et al., 2012; Dall et al., 2017; Ghaneialvar et al., 2018; Hu et al., 2018). Sca-1 can be used to subdivide bone-resident MSCs in mice into those with a predominantly adipogenic potential (Sca-1 positive) and a subset of cells with osteo-chondrogenic differentiation potential (Sca-1 negative) (Steenhuis et al., 2009; Lau et al., 2014; Ambrosi et al., 2017). Using Sca-1, we previously showed a multipotent MSC-subset expressing CD24 in addition to Sca-1 (i.e., Sca1-1+, CD24^+^), which shows tri-lineage (adipogenic, osteogenic, chondrogenic) differentiation potential in > 80% of individual cells (Ambrosi et al., 2017). Additionally, we reported a hierarchy of unidirectional specialization from this multipotent subset towards subpopulations of unilaterally committed adipogenic or osteo-/chondrogenic progeny (Ambrosi et al., 2017). Cells fate-committed to the adipogenic lineage loose expression of the surface marker CD24. A pre-adipocyte population expresses the transcription factor Zfp423 that can give rise to bone marrow adipocytes. Analogous cell populations expressing Zfp423 have been identified in white adipose tissue (WAT) and brown adipose tissue (BAT) (Gupta et al., 2012). Interestingly, the differentiation of Sca-1 positive MSCs towards CXCL12-producing cells has been shown to contribute to bone regeneration after injury by the regulation of MSC migration and induction of angiogenesis (Hu et al., 2016; Zhang et al., 2017). For the characterization of BMAT it is crucial to take into account that stromal cell subset appearance may differ between bone marrow cavities and the endosteal/cortical bone niche, presenting another possibility of phenotypically and functionally distinct mesenchymal cell subsets in different niches and anatomical localizations within the bone (Ambrosi et al., 2017; Craft et al., 2018; Pinho and Frenette, 2019). In light of our hypothetical distinction between healthy and unhealthy bone marrow adipocytes, it remains to be determined whether there are specific differentiation routes of adipogenesis-committed mesenchymal subsets into either regeneration-supportive or detrimental adipocytes, or whether pre-formed mature adipocytes change their phenotype in response to the microenvironment they encounter. The recent onset of single cell-based analysis technologies holds great promise to yield further insight into these processes (Baccin et al., 2020).

## 2 A brief overview on adipose tissues

**FIGURE 1:**
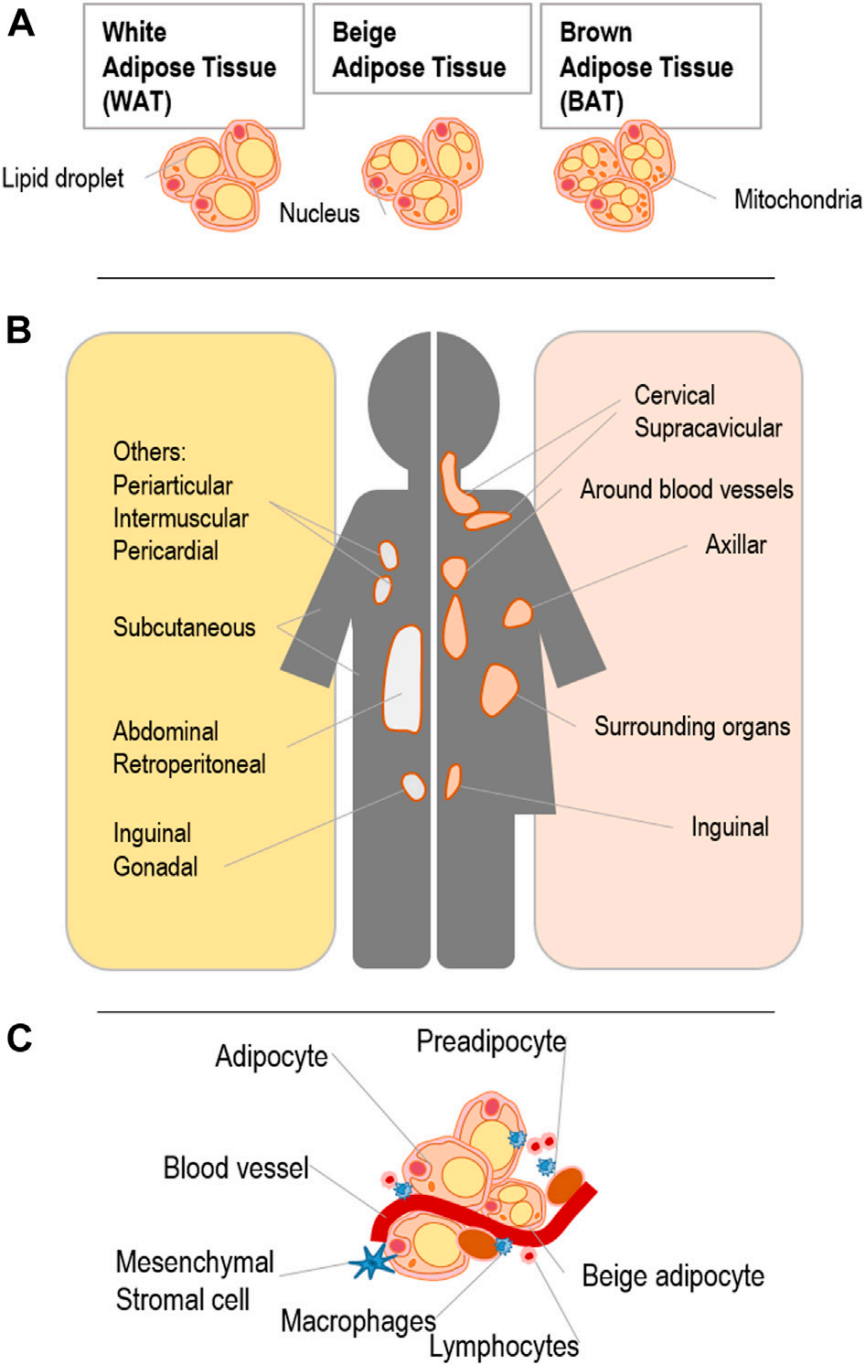
Anatomical locations of human WAT/BAT and beige adipose tissue with details on adipose tissue composition adapted from Lenz, et al. (2020): **(A)** Phenotype of WAT/BAT and beige adipose tissue differs in mitochondrial density and lipid droplet morphology. **(B)** Specific locations of each type of fat depot (brown or white) can be found throughout the body. Highlighted are the locations of adipose tissue surrounding organs, muscles and blood vessels. **(C)** Adipose tissue composition including adipocytes, immune cells, endothelial cells and mesenchymal stromal cells, which are all known to facilitate endocrine functions, energy storage and energy metabolism.

A key objective with respect to adipocyte-driven effects on bone regeneration is to determine which type of adipocytes resides within the bone marrow cavities. These adipocytes are key elements of metabolic and energy homeostasis (Konige et al., 2014). There are different types of adipocytes within the body with significant differences in gene expression as well as adipokine secretion patterns in distinct anatomical locations (Figure 1) (Ambrosi and Schulz, 2017; von Essen et al., 2017; Pond and Mattacks, 2003). In comparison to other adipose tissue depots, BMAT exhibits unique features differentiating them from the traditional depots of brown (BAT) and white adipose tissue (WAT).

### 2.1 White adipose tissue

WAT functions as energy storage organ featuring adipocytes with a single large, i.e., unilocular, lipid droplet. It provides thermal insulation and protects against mechanical stress in certain regions of the body, including hands, feet and buttocks (Luong et al., 2019). With triacylglycerols storing the bulk of chemical energy, white adipocytes have the ability to release free fatty acids under energy-demanding conditions. The process of lipolysis is a highly regulated biochemical pathway of triacylglycerol breakdown into non-esterified free fatty acids and glycerol [reviewed in (Duncan et al., 2007; Grabner et al., 2021)]. WAT is frequently identified using expression of biomarkers such as Peroxisome proliferator-activated receptor-γ (Pparg), Fatty acid-binding protein-4, (Fabp4, in older studies sometimes referred to as Adipocyte protein 2, aP2), Resistin (Retn), and the adipokine Leptin (Lep) (Cao et al., 2011).

### 2.2 Brown adipose tissue

BAT is a thermogenic adipose tissue that metabolizes fatty acids and glucose and dissipates the stored chemical energy in the form of heat (Basse et al., 2015). Thermogenesis depends on a high mitochondrial density and expression of Uncoupling protein 1 (Ucp1), and serves as protection from hypothermia and, potentially, obesity (Basse et al., 2015; Craft et al., 2019). In addition to UCP1 protein expression, BAT is also characterized by expression of marker genes such as Pparg, PR domain containing 16 (Prdm16) and Peroxisome proliferator-activated receptor γ - coactivator 1α (Ppargc1a, sometimes referred to as Pgc1a) (Bukowska et al., 2018).

### 2.3 Beige adipose tissue

In addition, intermediate forms of adipocytes have been described that represent a metabolically activated state between brown and white adipocytes (Lshibashi and Seale, 2010). These beige adipocytes, also known as brite (brown-in-white) adipocytes, are mainly found interspersed within WAT depots and are characterized by inducible expression of thermogenesis features, for instance in response to cold (Petrovic et al., 2010; Ikeda et al., 2018). Beige adipocytes are characterized by their ability to transdifferentiate from mature white adipocytes by switching from a uni to a multilocular lipid droplet morphology and by significantly increasing their mitochondrial content. The expression of thermogenic UCP1 is used as biomarker for both *bona fide* brown and cold-induced beige adipocytes (Craft et al., 2019; Hawkes and Mostoufi-Moab, 2019). The key function of UCP1, as a transmembrane protein in mitochondria, is the uncoupling of the mitochondrial proton gradient from ATP production, thereby enabling the release of energy from nutrient oxidation as heat (Craft et al., 2019). Aside from cold exposure, the process of browning of WAT is strongly regulated by external and environmental cues such as diet, exercise, exposure to drugs that act as PPARG agonists and tissue injury (von Essen et al., 2017; Abdullahi and Jeschke, 2016; Kaisanlahti and Glumoff, 2019). Browning is linked to increases in metabolic activity and flexibility as well as improved systemic insulin sensitivity, thereby presenting a new therapeutic strategy in the treatment of metabolic disorders like diabetes and obesity.

### 2.4 Bone marrow adipose tissue

Despite many morphological similarities, especially to white adipocytes, BMAT-resident adipocytes have been characterized as phenotypically distinct from other adipocytes in WAT or BAT and also beige adipocytes. It is currently unknown if a similar beneficial increase of insulin sensitivity and mitochondrial mass can be triggered in BMAT-adipocytes that parallels browning of white adipocytes. While BMAT seems to be largely incapable of browning, it is nonetheless known that bone marrow adipocytes (BMAds) exhibit similar sensitivity towards PPARG agonists than white adipocytes (Sulston et al., 2016; Craft et al., 2019; Suchacki et al., 2020). For instance, not all white adipocytes within WAT are similar and it is well established that important phenotypic differences exist between subcutaneous and visceral white fat depots. This in turn has significant implications for the pathological impact of these different regional types of adipocytes on metabolic health. Given that BMAds are widely distributed throughout different bones and the body, it stands to reason that this population of adipocytes also displays significant heterogeneity.

### 2.5 Unique characteristics of bone marrow adipocytes

In general, BMAds differentiate from MSCs within the bone marrow, which arise from a mesenchymal-osteogenic lineage and undergo lineage specialization involving a decision-making step between osteogenic and adipogenic lineage commitment (Liu et al., 2013; Chen et al., 2014; Ambrosi et al., 2019).

Interestingly, the potential role of adipogenic cells in bone has been further emphasized by using porous scaffolds seeded with human adipose tissue-derived mesenchymal stromal cells. In these scaffolds, adipocytes have been shown to contribute to osteoblast formation directly and to vascularization of bone defects as well as the induction of bone mineralization by the secretion of paracrine factors (Caetano et al., 2019). Thus, while bone marrow MSCs are phenotypically very similar to WAT-derived MSCs, they follow a distinct and more complicated differentiation trajectory to become mature adipocytes compared to WAT-MSCs which are presumably more lineage-committed to become adipocytes within WAT (Zhong et al., 2021). Therefore, BMAds can be considered developmentally distinct from other types of adipocytes within the body (Horowitz et al., 2017). In comparison to WAT and BAT, BMAT likely represents a specialized subtype of adipose tissue with close interactions to the skeletal and hematopoietic stem cell niches. Given that some level of marrow adipogenesis occurs early in life, some of this might not exert detrimental effects.

Recent studies have emphasized that BMAds are also clearly distinct from brown adipocytes and show very little propensity for expression of Ucp1 or cold-induced browning. In addition, their response to many physiological stimuli, including glucose homeostasis and insulin responses, is markedly different to brown adipocytes (Ambrosi et al., 2017; Suchacki and Cawthorn, 2018; Suchacki et al., 2020).

Regarding differences between BMAds, subtypes have previously been defined by their anatomical distribution within long-bones (Suchacki and Cawthorn, 2018). The initial report of these different subtypes dates back to the 1970s, where Tavassoli et al., described site-specific responses of BMAds in rabbits. In his work, red marrow contains a subset of disperse adipocytes, termed labile marrow adipocytes, which are characterized as potential interaction partners of the hematopoietic niche, whereas a subset of so-called stable adipocytes forms a coherent depot mainly consisting of mature adipocytes, which has been termed the yellow bone marrow (Tavassoli, 1976; Craft et al., 2018). These two different types of adipocytes are now termed as either residing within the regulated type of marrow adipose tissue (rBMAT), representing the labile adipocytes in red marrow or the constitutive bone marrow adipose tissue (cBMAT), representing the stable BMAds within yellow marrow regions.

Of note, cBMAT starts to appear during embryogenesis and/or in early childhood whereas the development of rBMAT is thought to occur later in life and during aging in particular (Li et al., 2018; Suchacki et al., 2020; Robles et al., 2021). The transplantation of red bone marrow to ectopic sites results in the formation of a hematopoietic bone marrow nodule at the site of transplantation. In contrast, the transplantation of yellow cBMAT results in the development of fat-filled bone marrow nodules (Tavassoli and Crosby, 1970). Biochemically, cBMAT is defined as a region of large, densely packed fat cells with unilocular vacuoles containing a higher proportion of unsaturated fatty acids (Scheller et al., 2015; Craft et al., 2018). cBMAT also expresses higher mRNA levels of stearoyl-CoA desaturases-1 and -2, which are important for the formation of monounsaturated fatty acids (Li et al., 2018). Recent data characterizes cBMAT as a last resort of energy storage which can be tapped during extreme conditions, such as prolonged starvation. CBMAT is able to take on the role of rBMAT when influenced by appropriate stimuli, especially in the young (Bigelow and Tavassoli, 1984). (Craft et al., 2018)

The adipocytes belonging to the rBMAT-type, on the other hand, are preferentially located in the proximal parts of the bone within red skeletal marrow regions with active hematopoiesis and harbor an increased number of small accessory lipid droplets in the peripheral cytoplasm (Robles et al., 2019; Scheller et al., 2019). The accumulation of rBMAT is strongly dependent on the lipodystropy gene Ptrf, which encodes for CAVIN1, a crucial component of lipid raft formation and insulin sensitivity (Li et al., 2018). These adipocytes are more sensitive towards skeletal remodeling and lipolysis (Scheller et al., 2015; Scheller et al., 2019). Furthermore, rBMAT can be found in niches harboring cell types like pericytes, hematopoietic cells and osteogenic cells. The hematopoiesis-active rBMAT regions share biochemical similarity with traditional depots of WAT as it displays a matching lipid profile, i.e., contains mostly saturated fatty acids (Scheller et al., 2015; Craft et al., 2019). Further, rBMAT is proposed to provide energy in the form of lipids/fatty acids to the surrounding environment (Scheller et al., 2019; Wolf et al., 2020). Another study shows that some adipocytes, but not within the regions of dense yellow marrow, which is mainly composed of cBMAT, act as energy-rich hubs within the stromal-reticular cell network of the bone marrow which can interact with its environment *via* indirect or direct cell-cell contacts (Robles et al., 2019). In terms of nutrient storage capacity, BMAT is characterized as storing a reduced quantity of glycogen and having a higher resistance towards insulin-stimulated lipolysis compared to traditional depots of WAT (Scheller et al., 2019), and some studies even propose that BMAT is insulin insensitive (Craft et al., 2019). However, recent evidence shows that BMAT within the distal regions of the bone is capable of responding to insulin and requires insulin to maximize lipid storage. Unlike these regions, the proximal rBMAT is rather known for its ability to respond to β-adrenergic signals, which induce lipolysis, remodeling of lipid droplets and release of fatty acids (Qiang et al., 2016; Scheller et al., 2019).

The development of BMAT progresses from early life, eventually filling up to 70% of the medullary space of some long-bones as early as by the age of 23 (Scheller et al., 2015; Hawkes and Mostoufi-Moab, 2019; Scheller et al., 2019). BMAT is further described to mimic some features of adipose tissue surrounding lymph nodes. An impact of perinodal adipocytes on the local immune response has been documented and a specific response towards cytokines derived from lymphoid cells is shown in adipocytes from adipose tissue near lymph nodes (Macqueen and Pond, 1998). Here, adipocytes provide energy in the form of lipids during immune activation (Craft and Scheller, 2017). Local immune cells release cytokines such as tumor necrosis factor α (TNFa) and interleukin 6 (IL6), causing the release of lipids from adipocytes and providing fuel for the local immune response. This is of particular importance during peripheral energy deficits, when immune cell reactions can be fueled without having to compete with other tissues (Craft and Scheller, 2017). Thus, an impact of BMAT on skeletal remodeling as well as immune cell activation and proliferation seems likely (Pond and Mattacks, 2003; Ambrosi and Schulz, 2017). The expansion of BMAT and conversion from red hematopoietic bone marrow towards yellow, adipocyte-rich bone marrow during various pathological conditions has been shown (Craft et al., 2019). Whilst the early accumulation of adipocytes seems to represent a physiological process, accumulation further increases during aging, and in obesity, where it can lead to pathological conditions.

### 2.6 Secretory role of bone marrow adipocytes

Adipokines are indispensable in many metabolic pathways (Vegiopoulos et al., 2017). They comprise genuinely adipocyte-specific proteins, like adiponectin and leptin, but also other signaling molecules, including bone morphogenetic proteins BMP4 and BMP7, which are members of the TGFb (transforming growth factor beta) superfamily, and which are not necessarily produced by adipocytes themselves but potentially by other adipose tissue-resident cell types. Other cytokines, like IL6 and TNFa, are also released from adipose tissues as signaling molecules that are known to regulate insulin sensitivity and energy homeostasis (Fasshauer and Paschke, 2003; Trujillo and Scherer, 2005; Fasshauer and Blüher, 2015; Schmidt-Bleek et al., 2016). The pathological impact of IL6 includes the induction of lipolysis, apoptosis and mitochondrial dysfunction, as well as steering lipotoxicity in the endoplasmatic reticulum (Abdullahi and Jeschke, 2016). Interestingly, IL6 released in response to injury appears primarily bone marrow-derived (Hardouin et al., 2016; Abdullahi et al., 2017).

It has been suggested that BMAT can contribute to the dysfunctional development of the osteogenic and hematopoietic niches by paracrine and endocrine signals (Ambrosi and Schulz, 2017). As an example, the adipokine hormone, adiponectin, which is secreted by adipocytes, is described to have contrasting effects on bone formation and turnover. Many reports show a pro-osteogenic role for adiponectin induced by increased osteoblast differentiation and activity (Berner et al., 2004; Williams et al., 2009; Huang et al., 2010; Jiang et al., 2011; Lecka-Czernik, 2012). Other studies characterize adiponectin as an anti-osteogenic factor, hampering osteoblast proliferation and differentiation (Lewis et al., 2021).

Adiponectin specifically secreted from adipocytes within the bone marrow niche promotes osteoblastogenesis and inhibits osteoclastogenesis, as well as inducing the expression of osteogenesis-related genes in animals and humans under healthy conditions. Further, adiponectin supports the proliferation, migration, and mineralization of osteoblasts. This balance is dysregulated during obesity and chronic inflammation, where either the loss of adiponectin or changes in the environment affect the signaling cascades triggered by adiponectin, such as mitogen-activated protein kinase (MAPK) of AMP-activated kinase (AMPK) signaling pathways. Here, controversial data on the bioactivity of adiponectin in the bone has been observed in animal and human studies (Lewis et al., 2021).

A complete lack of adipocyte derived endocrine factors is shown to exert detrimental effects on bone integrity (Scheller et al., 2015; Ambrosi and Schulz, 2017). Literature on the lack of adipocytes in the bone marrow microenvironment describes the alteration of both myeloid and lymphoid subsets and the dysregulation of hematopoietic stem cell mobilization (Wilson et al., 2018). In summary, whether bone marrow adipocyte accumulation poses a purely detrimental risk to bone health and regeneration could therefore be questioned. Since at least some BMAT arises early in life and produces important physiological signals, it stands to reason that a physiological role of bone-resident adipocytes may exist.

#### 2.6.1 Adipokines and their influence on bone marrow

To date, adiponectin is the most prominent systemically active endocrine factor for which BMAT has been described as a source (Falank et al., 2016). Adiponectin induces well-known beneficial effects on systemic insulin sensitivity, fat oxidation, inflammation and further exerts cardioprotective roles (Falank et al., 2016). During inflammation, adiponectin strongly influences the innate immune compartment, particularly M1-like and M2-like polarization of macrophages. Adiponectin downregulates pro-inflammatory cytokines such as such as TNFa, IL6 and monocyte chemotactic protein 1 (MCP1), thereby suppressing pro-inflammatory macrophage activation, while it promotes upregulation of IL10 and the polarization of anti-inflammatory macrophages (Luo and Liu, 2016).

During caloric restriction (anorexia nervosa), BMAT which paradoxically increases under these conditions, is a main source of adiponectin. Thus BMAT is able to exert systemic endocrine effects by contributing to increased serum adiponectin. (L Newton et al., 2013; Cawthorn et al., 2014). Systemic effects of adipokines secreted from BMAT are triggered through mechanisms including the production of inflammatory cytokines and bioactive lipids (Falank et al., 2016).

BMAT, as a source of secreted factors, has important effects on the bone compartment and its immune cell milieu. For instance, a forward feedback loop between adipogenesis and inflammation is proposed within bone. BMP2 is used clinically to support bone formation, but some studies have found that high doses of BMP2 are associated with potentially harmful side effects. Administration of BMP2 can increase inflammation and lead to excessive accumulation of adipocytes at the fracture site (Huang et al., 2018). Remarkably, however, this study also shows that immunomodulatory signals can influence adipocyte characteristics and their effects on the fracture healing process. Combined administration of BMP2 with IL6 promoted adipogenesis in the area of injury, but also simultaneously promoted osteogenic differentiation *in vitro* and bone formation *in vivo*. Although further analysis is required, these observations suggest that modulation of adipocyte phenotypes by appropriate signals may promote a beneficial or at least non-pathological profile of local adipocytes (Huang et al., 2018).

Like other physiological processes, the dosage as well as the controlled release of BMPs, adipokines and cytokines steer the impact on the endogenous regeneration cascade. BMP2 is currently used to aid bone fusion after fractures, emphasizing a beneficial effect of BMP2-driven adipocyte formation on bone metabolism (Schmidt-Bleek et al., 2016). BMAT is reported to produce higher levels of IL6 and TNFa in comparison to visceral adipose tissue. High pro-inflammatory signaling that is detrimental for regeneration (Bucher et al., 2019; Schlundt et al., 2019) on the other hand could be balanced by an upregulated adiponectin expression which in these cases has an anti-inflammatory function (Uchihashi et al., 2010; Hardouin et al., 2016). These findings altogether suggest that BMAT is an important regulator of the local microenvironment of bone tissue that contributes inflammatory signals to fine-tune the balance of pro-osteogenic, bone mass-increasing and bone-restorative signals.

Additional direct effects of BMAT on the myeloid and granulocyte cell lineages have been described, with adipocytes being not only unidirectionally influenced by immune cells, but rather being in an interdependent relationship with the immune cell compartment (Falank et al., 2016; Craft et al., 2019). Exemplarily, adipocytes are shown to be modulated by e.g., natural killer cells and adipocyte derived lipids can act as PPARG agonist to further stimulate increased adipocyte development. Adipocytes are able to exert local effects, for instance through bioactive lipids, but also systemic effects on energy balance and glucose/insulin homeostasis (Santoro et al., 2021). Local endocrine effects comprise the secretion of adiponectin and leptin from marrow fat with possible effects on osteoblast and osteoclast formation (Sulston and Cawthorn, 2016). The usage of recombinant Adenovirus to genetically modify adipose-derived stem cells in their adiponectin expression showed inhibitory features on adipogenesis and promoted osteogenesis in bone marrow mesenchymal stem cells (Yang et al., 2019). Other systemic effects are described, where osteoblast-secreted factors like osteocalcin and osteopontin are indirectly regulated by BMAT (Wu et al., 2014; Sulston and Cawthorn, 2016).

#### 2.6.2 Adipokines and their influence on site-specific BMAT regions

Site-specific secretion profiles are described for rBMAT and cBMAT. For example, the secretion of PAI-1 (plasminogen activator inhibitor-1) and cathepsin K, a protease involved in bone resorption, differs between both BMAT types, where PAI-1 is described to prevent bone loss by increasing bone formation and cathepsin K is indispensable for osteoclast-mediated bone resorption and remodeling (Jin et al., 2018; Dai et al., 2020). The site-specific paracrine effects of rBMAT and cBMAT are generally not well-studied but gain more and more importance as they may also differentially regulate bone maintenance and the healing outcomes of local bone defects (Sulston and Cawthorn, 2016).

The proximity of BMAT to the sinusoidal vasculature in the bone marrow cavity facilitates the uptake and storage of fatty acids and may help provide nutrients to bone lining cells. The increased dietary intake of poly-unsaturated fatty acids (PUFAs) has been shown to correlate with higher bone mineral density (BMD) and a reduced fracture risk (Bao et al., 2020). It should be noted that specific mechanisms of interaction of specific PUFAs with bone cells have been described. For example, omega-6 PUFAs are the biochemical sources of pro-inflammatory prostaglandins and are frequently discussed as negative determinants of fracture healing (Bao et al., 2020). It should be noted, however, that certain omega-6 derivates, such as the prostaglandins, exert distinct effects on osteogenic processes. The opposing inflammatory properties of prostaglandins are strongly dependent on their concentration. For example, low concentrations of prostaglandin E2 (PGE2) have been shown to induce bone formation whereas higher concentrations inhibit proliferation of osteoblasts *via* cAMP signaling (Ma et al., 1994; Baylink et al., 1996; Longo and Ward, 2016). The prostacyclin analog, Iloprost, has been shown to exert immunomodulatory effects driving a pro-regenerative milieu during bone fracture healing. In this case, the pro-inflammatory cytokine profile of T cells is downregulated and the mineralization capacity of mesenchymal stromal cells during osteogenic differentiation is enhanced (Wendler et al., 2019). Omega-3 PUFAs are shown to increase calcium resorption and bone collagen synthesis, thereby enhancing BMD (Bao et al., 2020). Bone marrow adipocytes are described to communicate with osteoblasts and immune cells by protein and lipid signals (Hubler and Kennedy, 2016; Robles et al., 2019) (Craft et al., 2018).

### 2.7 Marrow adipocytes: Subtypes, metabolism, and effects on the skeletal system

Ageing and obesity are two pathological processes accompanied by accumulation of adipocytes in bone marrow cavities and are therefore thought to contribute to the impairment of bone tissue regeneration. Ageing diminishes the function of the osteogenic lineage and is linked to loss of bone mass as well as increased fracture incidence. Our own research has shown that aged mice fed a high-fat diet display selective adipogenic lineage expansion of bone resident MSCs and that excessive numbers of adipogenic cells could be linked to impaired bone healing (Ambrosi et al., 2017). These results are in line with numerous studies correlating high levels of marrow adipocytes with impaired bone function. Further, BMAT is affected by pathological changes during obesity, which is also correlated with increased marrow adipogenesis and a decline of unsaturated fatty acid levels and impaired osteoblast/chondrocyte formation, as well as an enhancement of pro-inflammatory cytokines such as TNFa, IL6 and IL1b (Yu et al., 2017; Benova and Tencerova, 2020). It remains unclear whether marrow adipocytes directly compromise bone formation and strength, or only arise in response to bone loss or changes in the marrow microenvironment (Sulston and Cawthorn, 2016). The inverse relationship between marrow adipose tissue and BMD is somewhat debated and studies comparing different mouse strains have highlighted the fact that not all mouse strains with high levels of marrow adipogenesis also feature decreased BMD. For instance, the mouse strain C3HHe/J displays high levels of BMAT and high levels of BMD and trabecular bone mass (Fazeli et al., 2013). Of note, such strain-specific differences seem to be accompanied by differential expression of marker genes linked to brown adipogenesis and elevated mitochondrial metabolism, such as Ucp1 and Ppargc1a, in BMAT of the C3H/HeJ strain (Fazeli et al., 2013; Krings et al., 2012).

In patients, excessive amounts of adipose tissue, i.e., obesity, and adipose tissue deficiency in individuals with lipodystrophy, i.e., the selective or complete loss of all or most body fat, both lead to systemic metabolic dysfunction (Scheller et al., 2015; Suchacki et al., 2020). Patients suffering from congenital generalized lipodystrophy, including a lack of BMAT in bones develop osteosclerosis and skeletal cysts between the ages of 10 and 20 years, whereas (partial) lipodystrophy patients without loss of BMAT do not present this type of bone pathology [reviewed in (Scheller et al., 2015)]. However, an increase of BMAT in elderly people is clearly correlated with a decrease in BMD and increased fracture risk, suggesting that quality of BMAT may be more impactful than the amount of marrow resident adipocytes alone (Craft et al., 2019). Our own recent study using a murine tibia osteotomy model, found that injection of cells committed to the adipogenic lineage into the fracture site resulted in delayed bone healing as well as diminished hematopoietic recovery (Ambrosi et al., 2017).

These observations taken together challenge the widely held view of an unambiguously detrimental effect of BMAT accrual in general on osteogenic processes within the bone marrow, which has also been pointed out previously (Scheller and Rosen, 2014). In fact, other studies have described that marrow adiposity in younger individuals shows beneficial effects on the bone mineral content. It should be noted, however, that this effect was partially dependent on overall body fat, suggesting that BMAT in itself is not necessarily a determinant of mineral content [89]. Additionally, in type-2 diabetic patients (T2DM patients), where increased BMAT is observed, relative insulin deficiency or systemic insulin resistance and hyperglycemia have detrimental effects on bone formation and correlate with higher fracture rates (Gonnelli et al., 2015). T2DM patients with increased levels of BMAT are more susceptible to osteoporosis as well as certain fractures such as fragility-type fractures, classical osteoporotic fractures and osteoporosis-driven vertebral compression fractures (Warriner et al., 2011; Fazeli et al., 2013; Patsch et al., 2013; Carnevale et al., 2014). However, the higher fracture risk may be partially explained by the notion that patients may suffer from reduced coordination as a consequence of diabetes-related neurological impairment leading to a higher risk of falls (Farris et al., 2003; Luchsinger et al., 2007; Yau et al., 2013; Nadkarni et al., 2017).

The insulin responsiveness of BMAT and its role in systemic glucose homeostasis is not fully elucidated. Recent work suggests that BMAT is distinct from other white and brown adipocytes and features a marked resistance to insulin, resulting in lack of insulin-stimulated activation of the insulin signaling cascade and no induction of glucose uptake compared to other adipocyte types. It is also resistant to cold-induced glucose uptake and browning, but features markedly higher basal glucose uptake levels compared to other brown and white fat depots (Suchacki et al., 2020). Other studies have shown that rBMAT is sensitive to cold-mediated depletion, while cBMAT is not (Scheller et al., 2015; Robles et al., 2019). Of note, while beta-adrenergic agonists only activate lipolysis in rBMAT but not cBMAT, an activator of adenylate cyclase, which is also the downstream mediator of adrenergic signaling, was able to induce lipolysis in both types of BMAT, indicating that these two types of marrow fat feature distinct sensitivities to defined stimuli, but can non-etheless be activated to release their sored energy if treated with the appropriate signal (Scheller et al., 2019). During partial lipodystrophy, a redistribution of lipid storage in the body occurs, with BMAT being either not affected in certain lipodystrophies or similarly depleted by lipodystrophy-inducing mutations (Fretz et al., 2010; Scheller and Rosen, 2014). The biochemical characteristics of BMAT comprise its ability to store energy and to release nutrients, i.e., fatty acids, upon appropriate stimulation (Craft et al., 2019). The onset of cBMAT accumulation occurs during early infancy and is shown to be resistant to dissolution, suggesting that BMAT may contribute important functions to the bone microenvironment and exerts pathological effects only under certain (metabolic) conditions. Interestingly, reports showing detrimental effects of BMAT-accumulation on BMD mainly report negative correlations with rBMAT-expansion, which is mainly enriched in the proximal bone localities and more sensitive to metabolic stimuli such as cold and insulin (Scheller and Rosen, 2014; Scheller et al., 2015).

When specifically investigating distal accumulation of cBMAT, detrimental effects have not been shown and no unambiguous association of cBMAT accrual and loss of bone health has become evident from such analyses (Li et al., 1996; Miyakoshi et al., 1999; Scheller and Rosen, 2014; Scheller et al., 2015). One study shows that the extent of distal BMAT in mice, which broadly corresponds to cBMAT, remained stable from an early age of 12 weeks to later in life at 56 weeks of age. Amounts were also similar in strains with high and low overall BMAT, and had no correlation with impaired bone parameters (Scheller et al., 2015). Conversely, the expansion in proximal regions was more dispersed, corresponded to rBMAT and correlated with a typical age-related phenotype of reduced trabecular number and increased trabecular thickness (Scheller et al., 2015).

Human data on BMAT parallels the overall observations in mice (Ackert-Bicknell et al., 2009; Ambrosi and Schulz, 2017; Li et al., 2018). When focusing on rBMAT development, a key characteristic is its comparably high responsiveness to environmental and physiological stimuli. Factors positively correlated with the formation of rBMAT comprise ageing, obesity, and osteoporosis. BMAT accumulation in the proximal bone regions, that predominantly feature rBMAT, are also induced by health-promoting interventions, like caloric restriction and treatment with a class of anti-diabetic drugs, the thiazolidinediones (TZDs) (Li et al., 2018) [29]. Recently, adipogenesis in bone has been proposed as an emergency response promoting increased hematopoiesis by the secretion of stem cell factor (SCF; also known as KIT-ligand, KITL), and as being dependent on the local adipocyte type within the specific bone region. Through this mechanism, BMAT appears to promote maintenance of hematopoietic stem cells (Zhou et al., 2017).

Factors inhibiting rBMAT development include cold-exposure, exercise and extreme types of caloric restriction. When comparing lean and obese individuals, cBMAT seems to be unaffected regarding differences in lipid profiles, adipocyte numbers or size. Interestingly, while generally more inert compared to its counterpart, rBMAT, cBMAT is not fully resistant to some stimuli. It is moderately induced by TZD treatment and decreased by prostaglandin E2-receptor type 4 agonist treatment in ovariectomized rats (Li et al., 2018).

An inverse relationship of adiposity and bone volume is shown in various studies, while others show that adiposity does not necessarily lead to a decreased bone quality but may even increase bone density depending on the type of adipocytes being induced (Li et al., 2018). It is still unclear whether enhanced BMAT accumulation functions either as the primary cause of diminished bone regeneration or as a compensatory effect induced by environmental changes due to, for example, ageing, systemic inflammation, age-related pathologies and/or bone injuries (Schulz et al., 2016; Ambrosi and Schulz, 2017). The existence of two distinct types, regulated and constitutive BMAT, could provide an explanation for the apparently contradictory effects of metabolic regulation of marrow-resident adipocytes. Indeed, possible differences in bone/adipose tissue interaction could be dependent on the specific BMAT type. Further studies are required to determine if the two distinct types of BMAT do in fact represent either beneficial or pathological features during bone tissue repair and how each of them could be harnessed therapeutically (Seki et al., 2016; Craft et al., 2019). The notion of a distinct BMAT type developing locally during fracture repair is equally possible. The question would then be how to stimulate a predominantly beneficial BMAT phenotype in terms of regeneration support, immunomodulation, and metabolic activity.

## 3 Metabolic requirements of the bone healing cascade

**FIGURE 2:**
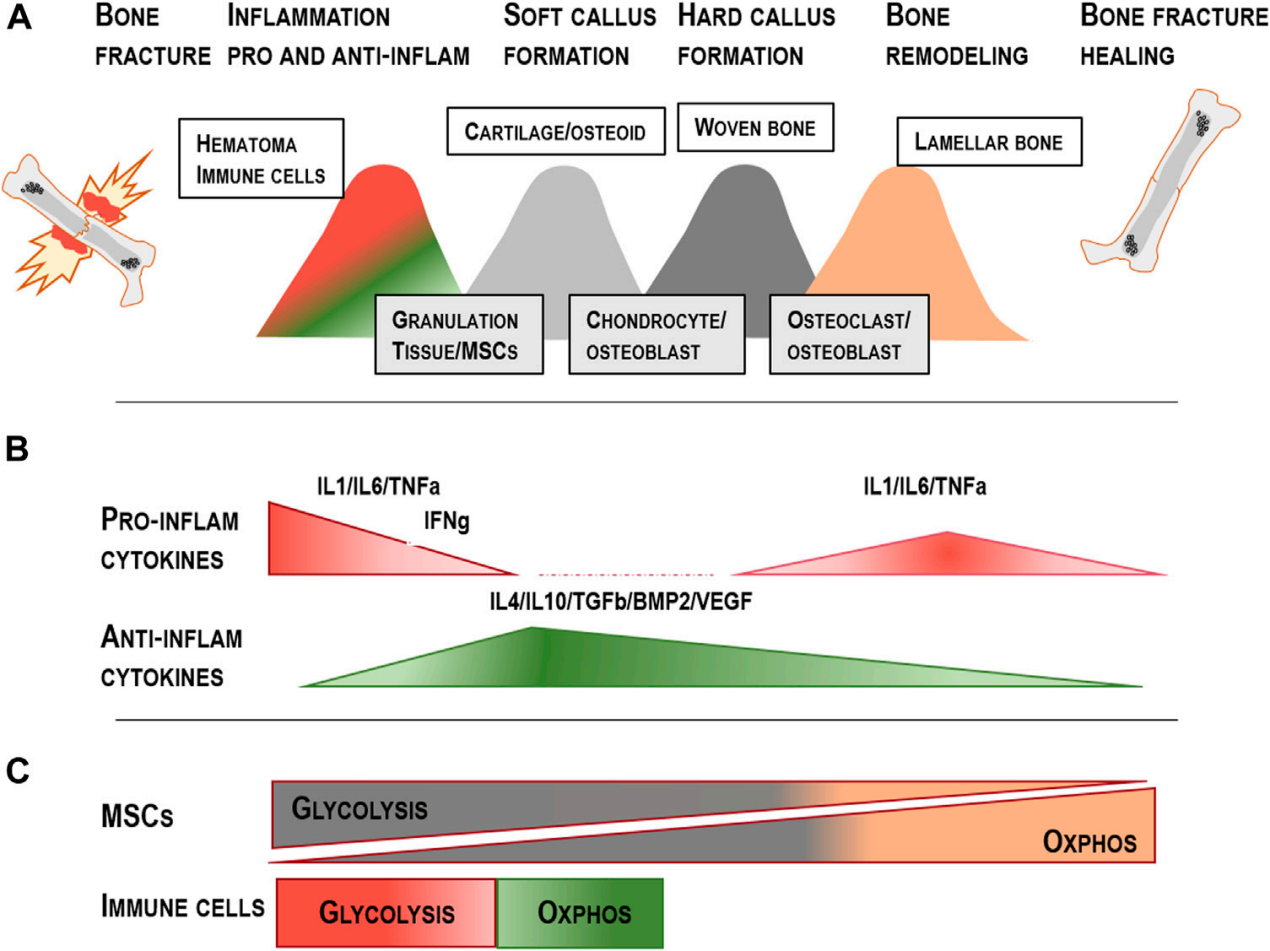
Overview of the five distinct fracture healing phases: **(A)** Stage 1: Hematoma formation and pro-inflammatory signalling; Stage 2: Anti-inflammatory hematoma; Stage 3: Soft callus development; Stage 4: Hard callus development; Stage 5: Remodelling of the newly formed bone; modified from Ono, et al. (2017) and Maruyama, et al. (2020). **(B)** Distinct cytokines affecting the pro- and anti-inflammatory phase during the early healing stage, callus formation and remodelling. **(C)** MSCs change their metabolic features from glycolysis towards OXPHOS during osteogenic differentiation. Monocytes, M1 macrophages and T cells prominently use the glycolytic pathways when differentiation and activation happens and change their metabolism towards OXPHOS when the anti-inflammatory phase is induced (Tregs/M2 macrophages) (Fretz et al., 2010; Scheller and Rosen, 2014; Loeffler et al., 2018).

Bone tissue can fully regenerate without excessive scar tissue formation. Successful healing, however, depends on the concerted participation of distinct cell populations and signaling cascades (Figure 2A). Despite this almost unique ability of scar-free healing, up to 10%–15% of patients show a delayed healing cascade or impairment of bone union (Ghiasi et al., 2017; Schlundt and Bucher, 2018).

Bone tissue repair is characterized by an initial hematoma formation and an acute pro-inflammatory reaction (Loeffler et al., 2018). Following bone injury, damage to blood vessels results in impaired delivery of nutrients to the injury site and reduction of cell migration. Moreover, hematoma-infiltrating immune cells further reduce the available oxygen and nutrients (Epari et al., 2008; Loeffler et al., 2018). The resulting hypoxia initiates the regenerative cascade, ultimately leading to the restoration of local nutrient and oxygen supply to the injured tissue. Immune cells are known to be critical pro-regenerative components of successful bone healing and are the first to infiltrate the early fracture hematoma. These infiltrating immune cells originate from the bone marrow and the disrupted blood vessels (Hoff et al., 2013). In addition to the trauma-induced influx, chemo-attractants comprising endogenous pro-regenerative lipid mediators (leukotrienes), as well as protein mediators (chemokines and cytokines) contribute to the infiltration of cells into the hematoma (Serhan et al., 2008).

The first cells infiltrating the fracture area are innate immune cells, initiating the pro-inflammatory response by cytokine secretion. Monocytes, macrophages, and T cell subsets undergo a critical metabolic shift from a quiescent state, where energy is provided preferentially through oxidative phosphorylation (OXPHOS), to a pro-inflammatory, activated state with increased energy demand (Figure 2C). As the early hematoma and callus is characterized by hypoxia, pro-inflammatory signal-producing monocytes and macrophages rely on anaerobic glycolysis to meet energy demands. Similarly, early callus T cells, such as CD4^+^ effector cells experience a reprogramming towards a predominantly aerobic glycolytic metabolism during differentiation [reviewed in (Loeffler et al., 2018)]. M1-like, pro-inflammatory macrophages, neutrophil granulocytes as well as CD8^+^ cytotoxic T cells from the adaptive immune system produce inflammatory signals, such as interleukin-1 (IL1) and TNFa. Subsequently, additional cells of the adaptive immune system, as well as CD34^+^ endothelial progenitors and MSCs are actively attracted towards the vicinity of the fracture gap, further driving the healing process. Thus, the adaptive immune response results in a high production of pro-inflammatory cytokines, activating and attracting MSCs for the facilitation of tissue regeneration (El-Jawhari et al., 2016; Schlundt and El Khassawna, 2018). During the initial inflammatory phase, cytokines also stimulate the essential induction of angiogenic processes and revascularization to achieve nutrient flow as well as improved cell migration capacity (Epari et al., 2008). Endothelial cells generally appear to prefer glycolytic substrate breakdown over aerobic OXPHOS, and during neovascularization, glycolytic flux is further enhanced (De Bock et al., 2013).

In bone and many other tissues, a pro-regenerative switch subsequently requires a transition from the pro-inflammatory milieu towards an anti-inflammatory signaling (Figure 2B). This change is induced chiefly by cytokines IL10, IL4 and TGFb, which are produced by immune cells, specifically macrophages and MSCs and can act as inhibitors of pro-inflammatory cytokine secretion (Mosser and Edwards, 2008; Schmidt-Bleek et al., 2009; Schmidt-Bleek et al., 2012; Heo et al., 2016; Loeffler et al., 2018; Lee et al., 2019; Liu et al., 2019; Han et al., 2022). In this context, MSCs can independently inhibit pro-inflammatory cytokine secretion, inducing immunosuppression and T cell inhibition by the release of nitric oxide (NO) in mice. In humans, T cell inhibition by indoleamine 2,3-dioxygenase (IDO) and prostaglandin E2 has been shown (Von Bergwelt-Baildon et al., 2006; Delarosa et al., 2009). Interestingly, prostaglandins, derived from the polyunsaturated fatty acid precursor arachidonic acid, are potent ligands for PPARG, the key pro-adipogenic transcription factor (Forman et al., 1995; Georgiadi and Kersten, 2012). The production of prostaglandins from poly-unsaturated omega-6 and omega-3 fatty acids has been shown to exert pro- and anti-inflammatory effects and to play a role in the regulation of bone formation (Scher and Pillinger, 2005; Bao et al., 2020). MSCs reside within the bone milieu under hypoxic conditions and strongly depend on glycolysis. However, as they specialize towards osteogenic differentiation, they undergo a pro-regenerative metabolic switch and induce TCA cycle enzymes and OXPHOS to generate ATP for osteogenic differentiation and matrix deposition during bone healing (Loeffler et al., 2018) (Figure 2C). By necessity, this process requires restoration of intact vasculature to provide the required nutrient and oxygen flux. In summary, these essential metabolic shifts pose the question whether targeting the metabolic features of MSCs and immune cells could represent a feasible approach to create pro-regenerative milieu to enhance the bone healing cascade under conditions of attenuate of defective bone healing.

Interestingly, PPARG is shown to promote glycolysis, glucose uptake and triglyceride synthesis by adiponectin induction (Nakamura et al., 2014). During adipogenesis, PPARG-driven effects depend on the induction of mitochondrial OXPHOS, which is likewise needed during osteogenic priming (Sparks et al., 2005; Ryu et al., 2013; Contreras-Lopez et al., 2020). This poses the question whether changing the metabolic features of MSCs and immune cells could represent a putative pro-regenerative starting point within the bone healing cascade.

## 4 Anti-diabetic drugs promote beneficial bone marrow adipocyte metabolism

Most diabetes drugs induce a beneficial metabolic and anti-inflammatory switch within adipocytes. This metabolic switch is linked to diverse effects on bone integrity, depending on the specific drug treatment. Aside from recombinant insulin-treatments, the most commonly used anti-diabetic drugs to date include sodium-glucose linked transporter 2 (SGLT2) inhibitors, TZDs, metformin, and regulators of the incretin effect, i.e., DPP4-inhibtors and incretin analogues (Cowie and Fisher, 2020).

The traditional antidiabetic drugs frequently display undesired side effects such as hypoglycemia, weight gain, cardiovascular complications and, in relation to bone health, increased fracture risk (Ammazzalorso et al., 2019). However, treatment of diabetes commonly requires drug treatments for extended periods of time, if not throughout life. It is therefore important to assess if the selective and time-limited use of anti-diabetic drugs could lead to beneficial effects on bone fracture healing, potentially driven by the accumulation of a BMAT type that supports regeneration. To date, the knowledge about the influence of BMAT on fracture healing is limited and, as discussed, there is some contradictory data on its impact on bone integrity.

To influence the accumulation of adipocytes in bone marrow, distinct approaches, including interventions targeting energy metabolism as well as certain drugs can be applied (Hegazy, 2015). Since the late 1990s, TZDs, such as pioglitazone and rosiglitazone, have been part of a widely used drug class for the management of T2DM. TZDs are thought to directly target the master regulator of adipogenesis, the nuclear receptor and transcription factor, PPARG, thereby ameliorating some aspects of insulin resistance (Thomas and Cherney, 2018; Ammazzalorso et al., 2019). Overall, contradictory side effects of TZDs are summarized in the literature (Berberoglu et al., 2010).

Among the know antidiabetic drug treatments, TZDs are known to most effectively influence BMAT accumulation and metabolism, as well as bone integrity (Suchacki et al., 2020). Notably, patients with diabetes show various skeletal disorders, including osteopenia and osteoporosis and enhanced fracture incidence. As a consequence of this metabolic disorder, insulin deficiency, insulin resistance or hyperglycemia may lead to abnormal cytokine and adipokine secretion profiles, thereby exerting detrimental effects on bone turnover and the bone microenvironment in general (Hegazy, 2015). Since TZDs act on multiple tissues aside from the skeleton, it is difficult to determine which effects on bone are due to the systemic impact as opposed to local, direct effects targeting either the osteogenic lineage directly or in a paracrine manner by acting on bone-resident adipocytes or even distinct subtypes of BMAT.

### 4.1 TZDs in the skeletal system

TZDs, such as rosiglitazone, act as PPARG-agonists and induce systemic insulin sensitivity, partially by the release of insulin-sensitizing adipokines from adipose tissue, and thereby improving systemic glucose homeostasis. PPARG-agonists also can decrease lipotoxicity by enhancing deposition of fatty acids into adipose tissue as triglycerides and thereby sequestering fatty acids away from other non-adipose tissues. In effect, the activation of PPARG as a key regulator of lipogenesis results in the reduction of fatty acids in circulation. Hereby, PPARG activation is regulating metabolic flexibility of adipose tissue under challenging metabolic conditions (Asterholm and Scherer, 2010; Sahebkar et al., 2014; Goodpaster and Sparks, 2017). Differential effects of TZDs on bone structure have been reported in the literature in clinical and pre-clinical models, either showing enhanced bone loss or few to no changes in parameters of bone health. A detailed study of the relationship between BMAT formation and bone health was previously conducted in different mouse strains and revealed that no unambiguous association between BMAT and BMD is evident. Specially, some strains, such as C57BL/6J displayed a remarkable and significant increase in BMAT after eight weeks of rosiglitazone but no effect on cortical thickness, the trabecular bone volume fraction and other parameters were found. Other strains responded with increased in BMAT that correlated with reduction of bone health parameters (Ackert-Bicknell et al., 2009). In healthy adult and aged mice without the context of diabetes, a 7-week rosiglitazone-exposure lead to a significant decrease in bone volume (Lazarenko et al., 2007). In young rats, the use of the TZD pioglitazone was equally detrimental to bone health and a 24-week treatment course resulted in reduction of trabecular and cortical BMD in the femur and enhanced bone resprtion (Kanda et al., 2017). Altogether these data highlight the importance in considering the local specifics of the interaction of the adipocytic lineage and bone health.

While previous reviews on the clinical aspects of bone health and TZD treatment generally agree that treatment is detrimental to bone health in diabetics, the extent has also been considered modest (Lecka-Czernik, 2010; Billington et al., 2015). Some studies indeed show that this relationship is either highly site-specific or depends on the type of TZD used, suggesting that the magnitude of PPARG-agonism is key to the effects on bone homeostasis. For instance, a 1-year randomized trial of the TZD pioglitazone in daily doses reported no effects on circulating bone turnover markers in patients with diabetes or impaired glucose tolerance. Nor did the study detect consistent decreases of bone mineralization, which was only observed in proximal regions of the femur but not, for instance, in the lumbar spine vertebrae (Grey et al., 2014). The effects of lobeglitazone, a newer member of the TZD family, were assessed in a double-blinded Korean study, and resulted in a mild but non-significant decrease of BMD, further supporting the notion that the bone-specific effect of PPARG-agonists needs to be evaluated carefully (Lim et al., 2017). In summary, it should be stressed that clinical data focus on long-term exposures to TZDs which indeed exert detrimental, if highly site-specific, effects on bone formation and resorption. Very little evidence was found regarding direct signaling-effects on osteogenic lineages and local, bone-resident adipocytes. As our previous discussion has shown, marrow-resident adipocytes display a pattern of metabolic regulation that is very distinct from other types and depots of adipocytes. It stands to reason that such differences may also translate in differential responses of BMAT to TZDs and the local interaction with cell types involved in bone maintenance and repair.

### 4.2 PPARG agonists–Enemies or allies of bone health?

In humans, the detrimental or beneficial impact of PPARG agonists, such as rosiglitazone, on bone seems to be highly dependent on the duration of therapy, with the adverse effects of rosiglitazone on bone structure only becoming apparent after approximately one year (Kahn et al., 2008). It is, however unclear whether TZD treatment or the underlying diabetes is responsible for the reduced bone quality and observed heightened fracture risk (Kahn et al., 2008).

On a cellular level, the sensitivity of osteoblast progenitors towards rosiglitazone decreases with age while the adipogenic lineage appears to become more sensitive to TZDs. This change in responsiveness coincides with increased levels of PPARG in the aged MSC pool which steer the rosiglitazone-driven increase in osteoclast progenitor development, leading to rosiglitazone-stimulated bone loss (Moerman et al., 2004). Rosiglitazone decreases the multipotency of MSCs by diminishing their ability to commit to different lineages, e.g., the osteogenic and adipogenic lineages (Moerman et al., 2004) thus defining detrimental bone loss induced by rosiglitazone treatment (Lazarenko et al., 2007).

Since the incidence of patients suffering from T2DM is steadily increasing, even in younger individuals, TZDs are more frequently prescribed. Thus, it is important to analyze TZD-based treatment and bone loss driven by anti-diabetic drugs such as rosiglitazone. Decreased osteoblast function or fatty acids that inhibit osteogenesis could also be a factor reducing bone quality (Kajimura et al., 2013; Wang et al., 2013).

**FIGURE 3:**
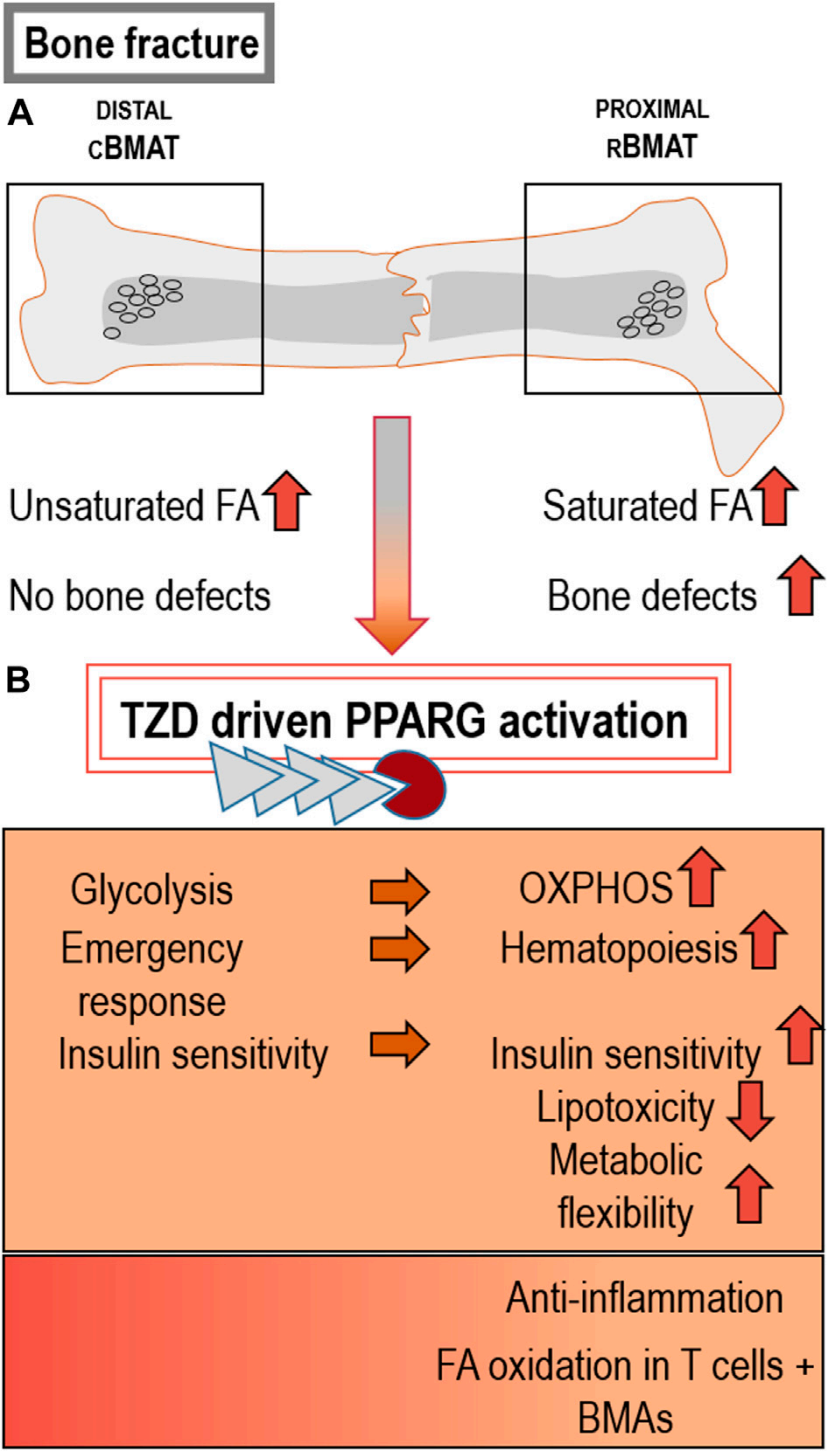
Different BMAT types and effects of PPARG activation on bone: **(A)** Distal cBMAT is characterized by higher amounts of unsaturated FA and its induction does not normally cause changes in bone health parameters, whereas proximal rBMAT negatively influences bone structure and regeneration. This detrimental impact on bone could be caused by its high levels of saturated FA (Lshibashi and Seale, 2010; Scheller et al., 2015; Sulston and Cawthorn, 2016; Bao et al., 2020). **(B)** TZD-driven PPARG activation and the resulting BMAT accumulation can induce beneficial metabolic features, such as higher OXPHOS rates, emergency haematopoiesis in response to injury, insulin sensitivity and lower lipotoxicity. Beneficial inflammatory effects can likewise result through the activation of PPARG by certain bioactive lipids, causing anti-inflammation and FA-oxidation in T cells and BMAds (Scher and Pillinger, 2005; Sparks et al., 2005; Asterholm and Scherer, 2010; Ryu et al., 2013; Sahebkar et al., 2014; Goodpaster and Sparks, 2017; Zhou et al., 2017; Chowdhury et al., 2018; Contreras-Lopez et al., 2020).

A previous study tested the effects of rosiglitazone on osteogenic differentiation and reported an enhanced adipogenic as well as osteogenic differentiation. Specifically, human mesenchymal stromal cells and other cell lines were either exposed to adipogenic or osteogenic differentiation conditions with and without addition of rosiglitazone. An RNAi-mediated knockdown of Pparg gene expression yielded similar results (Lee et al., 2013). Under these conditions, mineralization was enhanced during osteogenesis and cells expressed higher levels of osteogenic marker genes in response to rosiglitazone, providing some unexpected evidence for a controversial effect of TZDs during osteoblast differentiation and maturation. The authors conclude that the metabolic requirements during osteogenesis fluctuate and require an at least transient activation of mitochondrial energy metabolism to meet the energy demands of maturing osteoblastic cells. Thus, the activation of PPARG by rosiglitazone initially promoted mitochondrial metabolism and osteogenic differentiation (Figure 3B), but was later linked to mitochondrial dysfunction due to prolonged activation of PPARG and impaired cell survival due to onset of oxidative stress. To our knowledge this is one of the only studies to report robust induction of osteogenesis in response to TZDs and PPARG activation. These findings suggest that PPARG may pursue very distinct functions when targeting different cells contributing to the bone healing cascade.

Also, BMAT contains high levels of essential polyunsaturated fatty acids (PUFAs) in the form of triglycerides. TZDs can also bind these bioactive lipids, initiating processes relevant to osteogenesis, such as inflammation and marrow adipocyte formation (Abella et al., 2002; Calder, 2006; Devlin et al., 2010; Devlin, 2011). In addition, TZDs trigger high levels of fatty acid oxidation in adipocytes as well as in T cells (Figure 3B) (Chowdhury et al., 2018), thus offering a possible role of PPARG agonists in transducing signals from dietary fatty acids to immune cells involved in the bone healing cascade. This is important in metabolic diseases with underlying inflammatory components, but similarly in bone regeneration as part of the acute inflammatory injury response, i.e., during hematoma and early callus formation (Varga et al., 2011).

Prominent lipid mediators in bone, such as prostaglandins and leukotrienes, are well-known to regulate bone formation and inflammation (Wendler et al., 2019). These mediators are synthesized from omega-6 fatty acids and are crucial during bone healing. Recently, the prostaglandin 15-Deoxy-12,14-prostaglandin J2 (15d-PGJ2) has been described as an important endogenous ligand for PPARG, inducing an anti-inflammatory response involving PKA activation and inhibition of pro-inflammatory cytokines (Scher and Pillinger, 2005). The anti-inflammatory capacity of 15d-PGJ2 is also mediated by its specific inhibition of TNFa and IL1b production. For this inhibition, the involvement of mitogen-activated protein (MAP) kinases, NF-κB or IκB kinases have been proposed as mechanisms (Harris et al., 2002). Overall, the notion that omega-3 and omega-6 fatty acid species are potent endogenous activators of PPARG is well documented (Varga et al., 2011). Various stimuli, including dietary omega-3 PUFAs combined with pharmacological TZD interventions can induce a healthier adipocyte phenotype in the traditional fat depots of the body (Brejchova et al., 2020). It seems at least feasible to assume similar regulatory mechanisms of the signaling molecules in BMAT.

Only a limited number of studies have shed light on the process of lipogenesis in BMAT. However, the fatty acid composition of rBMAT, which contains higher levels of saturated fatty acids closely resembles the saturation profile of white adipocytes in other, localized depots of WAT (Figure 3A) (Griffith et al., 2009; Scheller et al., 2015; Hardouin et al., 2016). Conversely, cBMAT regions within the bone are characterized by higher levels of unsaturated fatty acids such as palmitoleate, oleate and a higher expression of stearoyl-CoA desaturase-1 (Scd-1), and thus display key distinguishing features compared to rBMAT and also adipose tissue depots (Scheller et al., 2015).

In BMAT, lipid unsaturation ranges from lower amounts in proximal rBMAT sites towards increased amounts in the distally located areas of cBMAT (Figure 3A) (Scheller et al., 2015). Fatty acids acting as PPARG ligands are described to steer lineage commitment of MSCs in bone (Rosen et al., 2009; Devlin et al., 2010).

In summary, the association of bone function and health with PPARG-activation, and the related interactions within a physiological context, are highly complex and remain only partially understood. There is some experimental evidence suggesting that TZDs, in their role as PPARG agonists, may not only promote formation of BMAT but additionally drive a bone-resident adipocyte phenotype that could exert beneficial physiological effects. For instance, the well-established immunomodulatory activity of adipocytes could be a potential mechanism of interaction between BMAT and immune cells during bone tissue repair and maintenance. However, there is a clear need for further research on this immune-BMAT interaction as a component of bone healing.

## 5 PPARG agonists as pro-regenerative cues that modulate inflammation during bone healing

**FIGURE 4:**
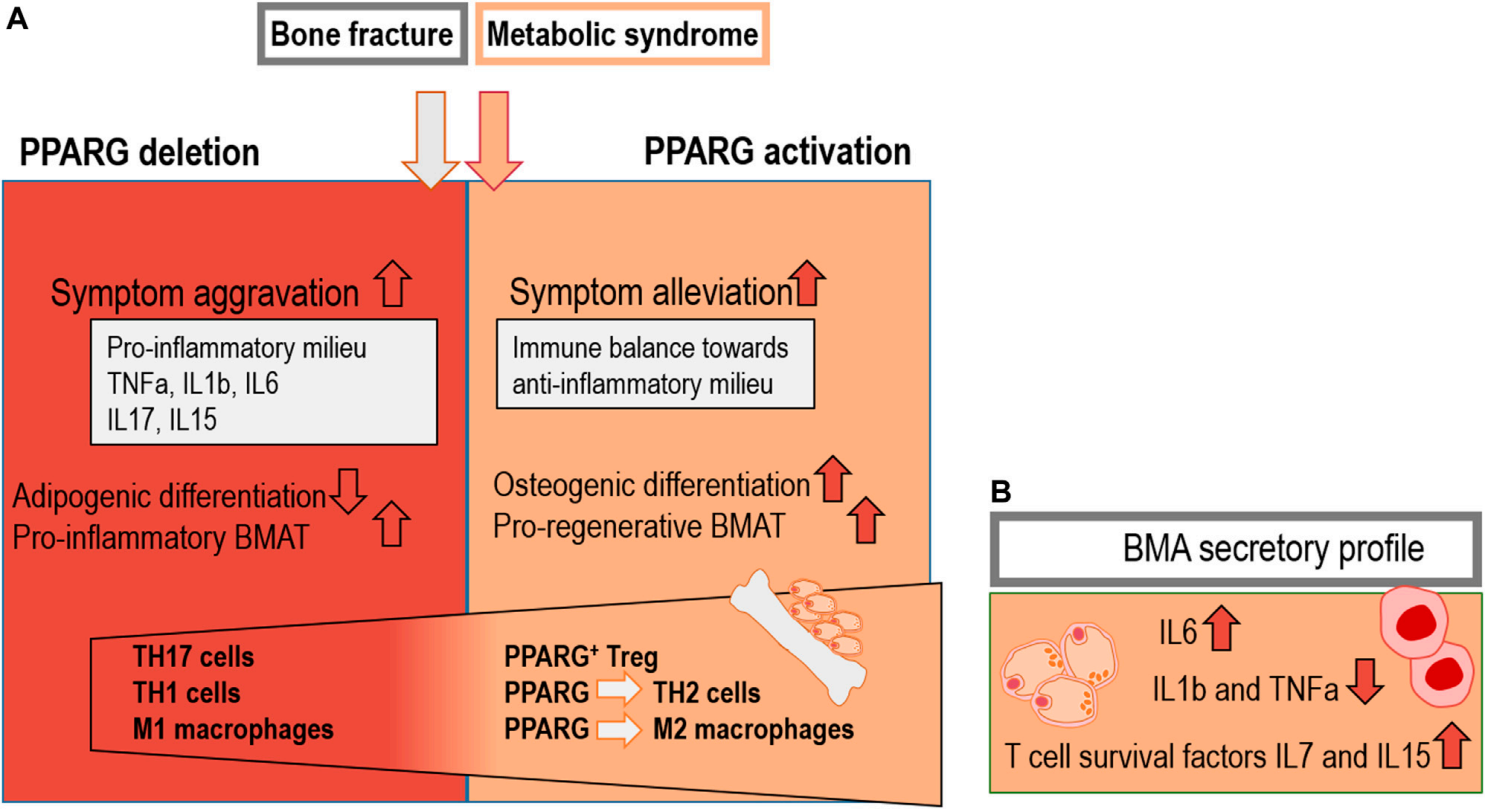
Combined effects of PPARG deletion and activation in metabolic syndrome and bone fracture: **(A)** PPARG deletion is shown to aggravate symptoms by the induction of pro-inflammatory cytokines such as TNFa, IL1b, IL6, IL17, and IL15, while reducing adipogenic differentiation. Inflammatory T cells such as TH17 and TH1 TH cells as well as M1 macrophages can be induced by PPARG deletion. PPARG-driven TH2 cell differentiation as well as the induction of PPARG+ Treg and anti-inflammatory M2 macrophages has been proposed (Hontecillas and Bassaganya-Riera, 2007; Hontecillas and Bassaganya-Riera, 2007; Miller, 2011; Angela et al., 2016; Heming et al., 2018; Menn and Neels, 2018; Miggitsch et al., 2019). **(B)** BMAds show elevated levels of IL6 secretion and well-known T cell survival factors IL7 and IL15 as well as reduced levels of pro-inflammatory IL1b and TNFa. These cytokines can have a potential impact on T cells in the bone marrow microenvironment (Laharrague et al., 2000; Miggitsch et al., 2019).

Without a balanced immune reaction during the initial bone healing phase, the regeneration cascade is disrupted. This in turn leads to detrimental effects which cause a delay in bone tissue repair as well as long-term consequences, such as non-union or sustained mechanical instability (Reinke et al., 2013). Other factors increasing the risk for delayed fracture healing are, for example, severity of the fracture, smoking, the use of anti-inflammatory drugs, malnutrition and conditions of impaired metabolism, such as diabetes (Holmes, 2017). PPARG is noted as an important regulator of bone resorption and formation, potentially by targeting signaling processes that control the inflammatory response, in addition to its well-established transcriptional activity during the adipogenic differentiation process (Rosen et al., 2009). BMAT harbors a specific inflammatory gene expression profile with higher levels of pro-inflammatory cytokines such as TNFa, IL1b and IL6, when compared to subcutaneous WAT. In addition, effector/memory T cell survival factors, such as IL7 and IL15, are expressed more highly in BMAT compared to subcutaneous WAT. IL7 and IL15 stabilize the pro-inflammatory milieu within the femur (Figure 4B; Miggitsch et al., 2019). Similar differences have been observed when comparing BMAT to murine epididymal WAT, a depot of visceral WAT, essentially confirming elevated levels of pro-inflammatory markers genes in BMAT (Liu et al., 2011). In contrast to the pro-inflammatory gene expression profile of BMAds, the secretory profile of BMAds in primary culture appears to comprise rather low amounts of IL1b and TNFa, whereas high levels of IL6 production were found (Figure 4B; Laharrague et al., 2000).

Pro-inflammatory conditions inhibit PPARG activity at the gene expression level and pro-inflammatory cytokines, such as TNFa, IFNg and IL1b, negatively regulate PPARG expression in the bone marrow, targeting e.g., macrophages (Nagy et al., 2013). (Takada et al., 2007) Conversely, TZDs could be used to shift the balance of the immune response towards an anti-inflammatory profile, as PPARG promotes an anti-inflammatory state of different immune cell subsets in the fracture setting (Figure 4A) (Heming et al., 2018). The following chapter will be structured in parts concerning different immune cell subsets. In detail, the specific influence of PPARG activation on regulatory T cells, T helper cells, type 1 and type 2 as well as macrophages will be elucidated.

### 5.1 Regulatory T cells

Successful bone healing is induced by regulatory T cells (Treg) and naïve adaptive immune cells that balance pro-inflammatory signals (Bucher et al., 2019). Consistent with this, co-culture of CD4^+^ T cells with PPARG-expressing Tregs resulted in a downregulation of IFNg production, which is beneficial for bone healing (Figure 4A; Hontecillas and Bassaganya-Riera, 2007). Additionally, Tregs are chondro-protective and induce endochondral ossification (Loeffler et al., 2018; Camacho et al., 2019). CD4^+^ Tregs likewise have the ability to limit inflammation in adipose tissues, thereby increasing insulin sensitivity (Hontecillas and Bassaganya-Riera, 2007; Ilan et al., 2010). Phenotype, function and accumulation of these specific Tregs in adipose tissue is highly dependent on the transcriptional activity of PPARG and Foxp3 (Hontecillas and Bassaganya-Riera, 2007; Kolodin et al., 2015). Thus, while it is clear that BMAT is highly distinct from other adipose depots, similar features could be relevant to influence detrimental pro-inflammatory processes within BMAT. In bone, Tregs are likewise shown to harbor features directly inhibiting bone resorption and controlling bone mass, as Tregs accumulate at sites of intense bone remodeling at the epiphysis of long bones. These Tregs influence bone formation depending on cell-cell contacts. Cell-cell contacts between osteoclasts and Tregs *via* CTLA-4, which is highly expressed on the surface of Tregs, is indispensable for the Treg-induced suppression of osteoclasts (Zhou et al., 2015).

### 5.2 T helper cells, type 1 and type 2

Human memory T helper cells reside within the bone marrow and can be activated independently of antigen encounters. Cytokines and chemokines produced by stromal cells mediate their survival and maintenance. The influence of BMAT accumulation on memory T cell subsets in bone is not well studied (Miggitsch et al., 2019). However, effector/memory T cell subsets are associated with pro-inflammation, whereas CD4^+^ memory T helper cells, especially CD4^+^ memory T helper 2 (TH2) cells, are associated with regulatory and anti-inflammatory features during bone repair. The induction of PPARG is required for the activation and proliferation of CD4^+^ naïve T cells and their development into CD4^+^ memory TH2 cells (Figure 4A). These CD4^+^ memory TH2 cells are known to be dependent on the PPARG-conditioned fatty acid uptake program (Angela et al., 2016; Miggitsch et al., 2019). In accordance with this, PPARG expression is predominantly associated with TH2 cells in humans and mice (which are beneficial for bone regeneration), thereby suggesting a potential additional effect of anti-diabetic TZDs on this specific immune cell subset. This is in line with other evidence that PPARG can resolve inflammation (Kostadinova et al., 2005). Deletion of PPARG in CD4^+^ T cells can cause severe overproduction of IFNg in response to IL12, emphasizing the impact of PPARG on the reduction of TH1 inflammatory responses (Figure 4A) (Menn and Neels, 2018; M. et al., 1998). Since PPARG in T cell subsets plays an essential role in controlling TH2 effector function, it has the potential to be a potent therapeutic target for steering bone healing by influencing this immune cell compartment. While long-term effects on bone through the systemic administration of PPARG agonists, for instance in the course of diabetes treatments, are broadly detrimental for bone homeostasis, a selective PPARG activation in bone-resident T cells could contribute to generating a pro-regenerative bone microenvironment. As such, the specific metabolic situations of bone homeostasis *versus* bone regeneration after a fracture need to be considered, as the requirements towards the niche, including the local adipocytes, may be distinct.

### 5.3 Macrophages

Macrophages with M1-or M2-like phenotypes have differential impact on bone regeneration (Gu et al., 2017). Pro-inflammatory M1-like macrophages are induced by low-grade systemic inflammation resulting from unbalanced cytokine release from immune cells as well as adipocytes (Sulston and Cawthorn, 2016). In bone, M2-like, anti-inflammatory macrophages contribute to tissue repair and are able to secrete cytokines that induce TH2 immune responses and other anti-inflammatory processes (Gu et al., 2017). More specifically, the activation of bone marrow-derived M2 macrophages by ligands of PPARG, PPARD, or the IL4-STAT6 signaling pathway induce an anti-inflammatory response, which is beneficial for bone formation (Figure 4A) (Ying et al., 2013; Menn and Neels, 2018). Since the ratios between M1/M2 macrophages and TH1/TH2 T cells are increased in obese WAT, the induction of an M2-macrophage phenotype in adipose tissue is linked to an improvement of this metabolic disorder by increasing insulin sensitivity (Kang et al., 2008; van den Berg et al., 2017; Brestoff and Artis, 2015). This scenario could likewise be associated with an alleviation of inflammation during bone healing. This is due to the M2-macrophage induction, which is important for the successful shift from pro- to anti-inflammation during regeneration of bone (Muñoz et al., 2020; Olmsted-Davis et al., 2021). Of interest is that M2 macrophage polarization is specifically induced by various BMAT-derived adipokines and this induction is detrimentally effected by age and disease (Reagan et al., 2021).

## 6 Concluding remarks

**FIGURE 5:**
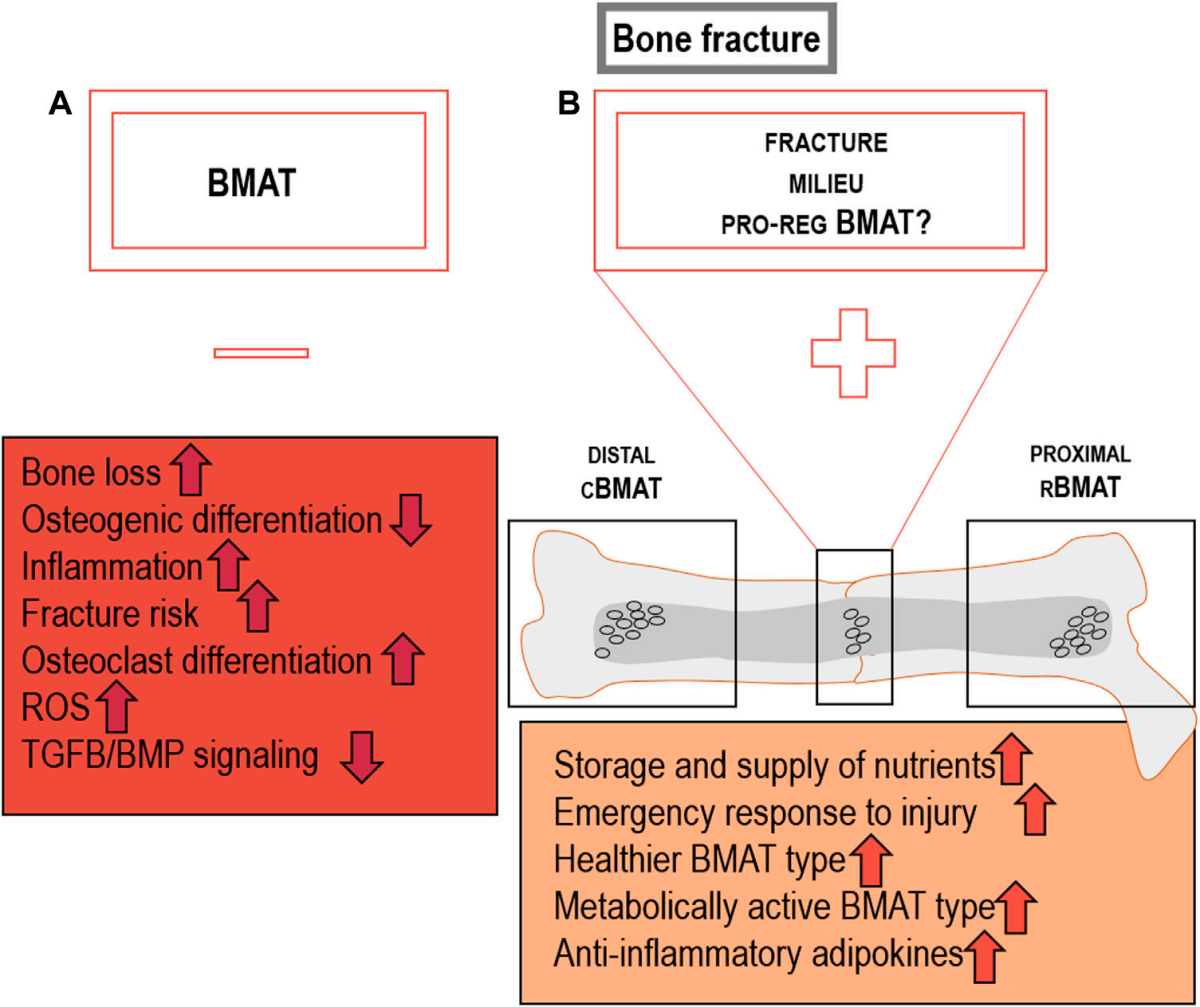
Detrimental BMAT characteristics are dependent on the location and milieu the BMAds: **(A)** BMAT may exert detrimental effects on bone by causing loss of bone mass and enhancing fracture risk by reducing osteogenic differentiation, TGFb/BMP signalling, while inducing osteoclast differentiation, ROS and inflammation (Lazarenko et al., 2007; Kahn et al., 2008; Ackert-Bicknell et al., 2009; Tang et al., 2009; Scheller et al., 2015; Falank et al., 2016; Lin et al., 2017; Ammazzalorso et al., 2019). **(B)** PPARG-driven, potentially pro-regenerative features of local BMAT at the fracture site which could include the storage and supply of nutrients to surrounding cells, representation as an energy hub for emergency responses, characterized as healthier, metabolically active, and anti-inflammatory BMAT type. BMAT could be recruited *de novo* or through plasticity of pre-exiting local adipocytes that convert into a regeneration-supportive phenotype.

Bone marrow adipose tissue is a poorly studied fat depot and its contribution to systemic metabolism as well as its role as a local contributor to the bone (marrow) niche remains only partially understood. Recently published evidence stresses that marrow adipocytes are distinct from other types of white adipocytes and have little, if any, resemblance to thermogenic brown adipocytes. However, the recognition of different types of bone marrow-resident adipocytes, cBMAT and rBMAT, in combination with the early-onset of marrow adipogenesis at a relatively young age, offers the intriguing possibility that this type of adipocyte is a key element of bone physiology, involved in the tissues energy homeostasis and a regulator of skeletal regeneration processes. Marrow adipocyte may target bone healing through different processes, including their insulin-sensitivity and metabolic activity. To this end, we review published evidence to explore the hypothesis that induction of marrow adipogenesis, for instance mediated by the insulin-sensitizing drug class of thiazolidinediones (TZD), in the context of skeletal repair may create a metabolically active type of marrow adipocytes as a pro-regenerative niche component (Figure 5B).

An important second aspect of this hypothesis is the timing of induction of marrow adipogenesis, which is clearly distinct from traditional inducers that promote marrow adipogenesis over a prolonged period. Accordingly, we review evidence to highlight a potential involvement of TZDs as agonist of the nuclear receptor PPARG and immune-modulation. To develop a strategy to enhance bone regeneration through targeting of PPARG-sensitive signaling and metabolic pathways, studying the combined effects of PPARG on bone and BMAT while also considering the immune reaction is of great importance. To this end, we have reviewed T cell-specific actions of PPARG as potential mechanisms relevant during bone regeneration. These processes have at present mainly received attention in the context of extra-skeletal adipose depots, i.e., the traditional depots of brown and white fat. However, the adaptive immune system, comprising distinct T cell subsets, has independently been shown to be essential for endogenous bone regeneration.

The presence and impact of metabolically distinct BMAT subtypes during bone regeneration is unknown and remains to be investigated in future studies. In summary, the characterization of BMAT subtypes within the bone compartment and their specific impact on bone injury repair and regeneration may require a more nuanced definition of BMAT functions, highlighting site-specific differences of BMAT as well as the impact of different metabolic and pathological conditions that BMAT is exposed to. The concept of adaptive changes in BMAT characteristics resembles the immune response during healing of bone, where the plasticity of cell types and inflammatory cues is highly dependent on the surrounding environmental milieu and the stage of bone healing.
